# Patient Factors Impacting Perioperative Outcomes for T1b-T2 Localized Renal Cell Carcinoma May Guide Decision for Partial versus Radical Nephrectomy

**DOI:** 10.3390/jcm12010175

**Published:** 2022-12-26

**Authors:** Yash B. Shah, Rishabh K. Simhal, Kerith R. Wang, Hanan Goldberg, Costas D. Lallas, Thenappan Chandrasekar

**Affiliations:** 1 Sidney Kimmel Medical College, Thomas Jefferson University, Philadelphia, PA 19107, USA; 2 Department of Urology, Thomas Jefferson University, Philadelphia, PA 19107, USA; 3 Department of Urology, State University of New York Upstate, Syracuse, NY 13210, USA; 4 Sidney Kimmel Cancer Center, Thomas Jefferson University, Philadelphia, PA 19107, USA; 5 Department of Urology, University of California Davis, Sacramento, CA 95616, USA

**Keywords:** renal cell carcinoma, partial nephrectomy, radical nephrectomy, patient outcomes, perioperative complications, surgical decision-making, patient comorbidities, NSQIP

## Abstract

There remains debate surrounding partial (PN) versus radical nephrectomy (RN) for T1b-T2 renal cell carcinoma (RCC). PN offers nephron-sparing benefits but involves increased perioperative complications. RN putatively maximizes oncologic benefit with complex tumors. We analyzed newly available nephrectomy-specific NSQIP data to elucidate predictors of perioperative outcomes in localized T1b-T2 RCC. We identified 2094 patients undergoing nephrectomy between 2019–2020. Captured variables include surgical procedure and approach, staging, comorbidities, prophylaxis, peri-operative complications, reoperations, and readmissions. 816 patients received PN while 1278 received RN. Reoperation rates were comparable; however, PN patients more commonly experienced 30-day readmissions (7.0% vs. 4.7%, *p* = 0.026), bleeds (9.19% vs. 5.56%, *p* = 0.001), renal failure requiring dialysis (1.23% vs. 0.31%, *p* = 0.013) and urine leak or fistulae (1.10% vs. 0.31%, *p* = 0.025). Infectious, pulmonary, cardiac, and venothromboembolic event rates were comparable. Robotic surgery reduced occurrence of various complications, readmissions, and reoperations. PN remained predictive of all four complications upon multivariable adjustment. Several comorbidities were predictive of complications including bleeds and readmissions. This population-based cohort explicates perioperative outcomes following nephrectomy for pT1b-T2 RCC. Significant associations between PN, patient-specific factors, and complications were identified. Risk stratification may inform management to improve post-operative quality of life (QOL) and RCC outcomes.

## 1. Introduction

Renal cell carcinoma (RCC) is associated with over 70,000 diagnoses and 14,000 deaths annually in the United States [1,2,3]. Incidence increased by 3–4% annually over the past 25 years, partly due to stage migration from improved diagnostic imaging [4]. Although radical nephrectomy (RN) was previously standard for all renal tumors, earlier detection and improving robotic technologies have precipitated growing utilization of partial nephrectomy (PN) [5,6,7].

Nephron-sparing surgery offers long-term benefits including reduced chronic kidney disease (CKD) and associated cardiovascular or metabolic sequelae [8,9,10]. Conversely, retrospective studies demonstrate increased perioperative complications with PN, often attributed to its technical complexity [1,11]. Importantly, these results may be confounded by institutional variations in surgical technique, demographics, and selection bias. Accordingly, the literature is largely inconclusive; a separate analysis of cT2 masses found comparable perioperative complications and survival [11]. European Organisation for Research and Treatment of Cancer trial 30904 demonstrated oncologic equivalence, though perioperative complications including bleeding, urinary fistulae, and reoperations were increased with PN; however, enrollment concluded in 2003 and techniques have since improved, particularly with the advent of minimally invasive surgery (MIS) [12,13,14].

Presently, PN is recommended for T1a RCC, while RN remains customary for locally advanced T3 tumors [15,16]. However, there remains significant equipoise for T1b-T2 disease, where decisions are largely based on gestalt. The National Comprehensive Cancer Network recommends either PN or RN, while various associations offer conflicting practice guidelines [17,18]. Three scores, the RENAL nephrometry score, PADUA, and c-index are commonly used to facilitate decision-making based on anatomic factors, but these systems do not account for non-anatomic patient-specific factors such as comorbidities.

Patient- and tumor-specific characteristics, including demographics, comorbidities, tumor size, and histopathology, were previously demonstrated to impact survival following PN versus RN for T2 masses [6,8]. Although risk factors affecting long-term outcomes have been compared, their predictive value for near-term complications remains unclear. Ultimately, clinicians must consider whether increased perioperative morbidity offsets the advantages of PN, and whether appreciating risk factors can allow prediction and mitigation of such morbidity. Considering the equipoise in this disease space, updated guidance would improve localized RCC management [13,19].

The National Surgical Quality Improvement Program (NSQIP) database captures standardized data on baseline characteristics and perioperative outcomes from over 600 hospitals [20]. By utilizing NSQIP and its new Nephrectomy Participant Use Data File (PUF), this study captures a modern, cross-institutional cohort to elucidate nephrectomy outcomes and predictors of perioperative morbidity. We hypothesized that patient-specific factors associated with specific perioperative outcomes would guide PN versus RN decision-making for T1b-T2 RCC in cases of oncologic equivalence.

## 2. Materials and Methods

### 2.1. Study Population

Within the 2019–2020 NSQIP and Nephrectomy-Targeted data, 8404 RCC patients were identified using the International Classification of Diseases, Tenth Revision (C64, C64.1, C64.2, C64.9). Upon extracting 2165 stage T1b and T2 patients, 16 patients were excluded given pre-operative sepsis, 24 for contaminated wounds pre-operatively, and 31 for node-positivity or metastasis. Ultimately, 2094 patients were included.

### 2.2. Description of Covariates

Variables of interest included age, race, ethnicity, pathologic stage, and pre-operative diabetes, smoking, respiratory conditions, cardiovascular diseases, chronic steroid use, bleeding disorders, and creatinine. Complications of interest included surgical site infections (SSIs); urinary tract infections (UTIs); sepsis; wound dehiscence; cardiovascular, pulmonary, and renal events; venothromboembolism (VTE); bleeding events within 72 hours requiring transfusion; and urine or lymphatic leaks, fistulae, or obstructions. NSQIP acute renal failure, defined as new post-operative dialysis requirement, was denoted “renal failure requiring dialysis”, while NSQIP progressive renal insufficiency, defined as creatinine increase >2mg/dL without dialysis, was denoted “acute kidney injury (AKI)”. Surgical variables including stent and drain placement, perioperative antibiotics, VTE prophylaxis, and surgical approach were described, as were quality measures including operative time, length of hospital stay (LOS), reoperations within 30 days, and readmissions within 30 days.

### 2.3. Statistical Analysis

Descriptive statistics for demographics and complications were compared by nephrectomy type, surgical approach, and comorbidities using Fisher’s exact test and Pearson’s chi-square test. Multivariable logistic regression hazard models were generated to report odds ratios (ORs) comparing complication rates between PN and RN following correction for associations (demographics, comorbidities, pre-operative measurements, and surgical approach) deemed significant on univariate analysis or clinically relevant a priori.

All statistical tests were two-tailed. *p* < 0.05 was considered statistically significant. Statistical analyses were performed using GraphPad Prism 9.0.2 (San Diego, CA, USA) and SPSS 28.0.1 (IBM Corp., Armonk, NY, USA).

## 3. Results

### 3.1. Demographics

Overall, 816 (39.0%) patients underwent PN while 1278 (61.0%) underwent RN. Demographics and baseline characteristics were compared between these two groups (Table 1). T1b patients received PN more commonly than T2 patients (*p* < 0.001). PN patients were younger (60.22 vs. 62.32, *p* < 0.001) and more commonly male (68.1% vs. 63.8%, *p* = 0.044). Respiratory or cardiovascular disease history was similar between the two groups, although more PN patients had diabetes (26.4% vs. 20.6%, *p* = 0.002). Patients on dialysis before surgery typically received RN (*p* < 0.001).

### 3.2. Outcomes following PN versus RN

Within 30 days of the initial operation, 37 (1.8%) patients required reoperation, while 117 (5.6%) patients were readmitted (Table 2). The PN and RN cohorts had comparable reoperation rates (2.1% vs. 1.6%, *p* = 0.380), although there were more readmissions following PN (7.0% vs. 4.7%, *p* = 0.026). LOS was comparable (*p* = 0.472). PN operations generally took longer, lasting an average of 198.45 ± 72.81 m versus 155.24 ± 72.75 m for RN (*p* < 0.001).

Importantly, bleeds occurred more commonly with PN (9.19% vs. 5.56%, *p* = 0.001), as did urine leaks or fistulae (1.10% vs. 0.31%, *p* = 0.025). Moreover, renal complications were largely more common following PN, including AKI (1.10% vs. 0.39%, *p* = 0.051) and renal failure requiring dialysis (1.23% vs. 0.31%, *p* = 0.013). Infectious, cardiovascular, pulmonary, and VTE occurrences were comparable. Although perioperative antibiotic use was similar, VTE prophylaxis differed, as mechanical prophylaxis alone was common in PN while more RN patients received combination prophylaxis (*p* < 0.001).

### 3.3. Impact of Surgical Approach

Given the expanding indications of MIS, complication rates were also compared by surgical approach (Supplementary Table S1). Open cases saw more VTE (2.19% vs. 0.19%, *p* = 0.025), renal failure requiring dialysis (1.82% vs. 0.26%, *p* < 0.001), bleeds requiring transfusion (16.61% vs. 3.56%, *p* < 0.001), urine leaks or fistulae (1.28% vs. 0.39%, *p* = 0.050), readmissions (8.03% vs. 4.72%, *p* = 0.005), and reoperations (2.92% vs. 1.36%, *p* = 0.023).

### 3.4. Predictors of Perioperative Events

Univariate analysis uncovered comorbidities associated with particular complications (Table 3). Upon multivariable analysis (MVA), with nephrectomy type, approach, and relevant comorbidities included as covariates, patient-specific predictors were uncovered (Table 4). MVA was repeated for the PN and RN cohorts separately to further clarify risk factors (Supplementary Tables S2 and S3).

#### 3.4.1. Infectious

SSIs and sepsis events were not predicted by any patient factors. Wound dehiscence was associated with pre-operative renal failure (OR 175.494, *p* < 0.001). Notably, pneumonia was predicted by COPD (OR 5.789, *p* < 0.001) and bleeding disorder (OR 6.596, *p* < 0.001). The occurrence of UTIs was only predicted by hypertension (OR 3.012, *p* = 0.047). Nephrectomy type did not influence infectious events.

#### 3.4.2. Cardiovascular

Although diabetes was predictive of MI (OR 4.988, *p* = 0.013), no patient factors were predictive when cardiovascular events—including MI, CVA, and cardiac arrest—were combined as a single outcome. Nephrectomy type did not predict any cardiovascular events.

#### 3.4.3. VTE

On MVA, VTE was predicted by steroid use (OR 4.245, *p* = 0.024) and open surgery (OR 2.480, *p* = 0.025). Specifically, DVTs were predicted by open surgery and steroid use, while PEs were only predicted by COPD. Procedure type did not affect VTE rates.

#### 3.4.4. Bleeding

PN was associated with an increased risk of bleeds requiring transfusion (OR 1.524, *p* = 0.023). Moreover, bleeds were predicted by open surgery (OR 5.070, *p* < 0.001), dialysis (OR 3.825, *p* = 0.004), and bleeding disorder (OR 3.105, *p* = 0.002).

Open surgery conferred risk following both PN (OR 5.327, *p* < 0.001) and RN (OR 4.662, *p* < 0.001). Diabetes (OR 2.032, *p* = 0.009 vs. OR 1.001, *p* = 0.997) and hypertension (OR 1.956, *p* = 0.039 vs. OR 1.196, *p* = 0.528) only predicted bleeds in the PN cohort. Pre-operative dialysis (OR 4.709, *p* < 0.001) and bleeding disorder (OR 4.216, *p* = 0.001) only increased risk in RN patients.

#### 3.4.5. Renal

Renal failure requiring dialysis was more common following PN, although this lost significance as a predictor following multivariable adjustment for surgical approach (OR 2.811, *p* = 0.066).

Pre-operative acute renal failure was a strong predictor within the PN cohort (OR 36.961, *p* = 0.016). No pre-operative renal failure patients receiving RN experienced this complication. Similarly, open surgery was highly predictive within the PN cohort (OR 7.323, *p* = 0.014), but not within the RN cohort (OR 3.598, *p* = 0.206).

Hypertension predicted AKI on MVA (OR 7.689, *p* = 0.049). After covariate adjustment, PN patients appeared slightly more prone to experience AKI (OR 2.740, *p* = 0.098), although this did not reach statistical significance.

#### 3.4.6. Urine and Lymph Leak

Although urine leaks or fistulae occurred more commonly in PN patients, this was not predicted by nephrectomy type following multivariable adjustment for surgical approach (OR 3.000, *p* = 0.073). Open surgery did not demonstrate different effects with PN (OR 2.482, *p* = 0.182) versus RN (OR 4.067, *p* = 0.164). Ureteral obstructions were not associated with any risk factors.

Dialysis (OR 6.680, *p* = 0.005) and renal failure (OR 12.369, *p* = 0.040) were predictive of lymphatic leaks on MVA, while procedure type was not.

#### 3.4.7. Readmissions and Reoperations

PN was predictive of increased 30-day readmissions (OR 1.568, *p* = 0.024). Readmissions were also predicted by open surgery (OR 1.511, *p* = 0.019), renal failure (OR 12.734, *p* = 0.017), chronic steroid use (OR 2.511, *p* = 0.020), and COPD (OR 2.646, *p* = 0.010). Interestingly, CHF increased readmission risk in PN patients (OR 14.199, *p* = 0.010); no RN patients with CHF required readmission. Conversely, steroids (OR 4.506, *p* = 0.001), bleeding disorder (OR 5.612, *p* < 0.001), and COPD (OR 5.403, *p* < 0.001) predicted readmissions in RN patients alone.

Reoperations were predicted by pre-operative renal failure, dialysis, steroid use, and bleeding disorders on MVA, while nephrectomy technique did not affect reoperation rates.

## 4. Discussion

This study analyzed a representative population of 2094 localized T1b/T2 RCC patients receiving nephrectomy from 2019–2020. The NSQIP Procedure-Targeted PUFs have never been previously utilized to describe urology-specific quality metrics, and the literature lacks a population-based comparison of the competing short-term risks and benefits of PN and RN [11]. Previous studies of smaller cohorts were limited in generalizability or stratification by case-specific factors.

Patients with pre-existing diabetes, hypertension, or cardiovascular risk often undergo nephron preservation. Associated benefits should be weighed with risks of perioperative complications following this technically complex procedure. Understanding patient-specific factors modulating that risk may facilitate treatment choice.

Demographic differences between our cohorts, including increased PN utilization in younger males with lower tumor stages, are supported by previous studies [6,9]. The disparity in operative time, which is well-described, serves as a surrogate measure demonstrating the increased technical complexity of PN. Differences in perioperative morbidity are also supported [12,14,21]. However, no studies have identified specific patient populations in which perioperative complications were actually relevant, hence precluding real-world application. A study of 2011 NSQIP data found that readmissions following RN were not predicted by demographic factors, but comorbidity data were not analyzed [22].

Considering the decrease in nephrectomy complication rates over time, along with recent improvements in MIS, an updated analysis is required [5]. Robotic PN demonstrates reduced complications, bleeding, and readmissions than open PN [5,23,24]. Our modern cohort corroborated this trend, demonstrating that increased adoption of MIS may expand indications for PN while mitigating its associated peri-operative risks.

This study suggests significant associations between PN and the following four complications: bleeding, renal failure requiring dialysis, ureteric leaks or fistulae, and readmissions. This understanding may either inform preventative measures to reduce risk for PN patients or guide surgeons towards RN in cases where these complications would be undesirable. Notably, within the PN cohort, MIS was associated with fewer cases of renal failure requiring dialysis and urine leaks. Certainly, MIS was generally safer and more common with PN than RN, but it is possible that this association also reflects surgeon selection bias. Notably, adjustment for any patient-specific comorbidities lacked the same effect, indicating that these two complications are still important considerations, as patients with anatomically complex tumors are likely to continue to receive open surgery.

Ultimately, it appears that although PN offers several benefits for localized RCC patients and has low complication rates, those with certain baseline characteristics may fare better with RN. Clinician awareness of differential risks following PN versus RN can facilitate patient-specific treatment choice based on values surrounding post-operative QOL. For instance, patients in whom bleeds would be particularly undesirable (e.g., bleeding disorder or Jehovah’s witness patients) may be poorer candidates for PN. Those interested in improved short-term QOL may prefer RN to avoid readmission.

Although renal failure requiring dialysis was significantly increased for PN patients, even after multivariable correction for baseline renal function, this may be attributable to selection bias. Patients with low preoperative renal function or CKD are frequently recommended PN considering their higher risk for end stage renal disease (ESRD) progression. The NSQIP database does not include pre-operative CKD, and the available pre-operative renal failure variable only captures acute declines in renal function within 24 h prior to the index operation [16,25]. Our PN cohort may contain a disproportionate amount of baseline CKD patients. Recent findings suggest that such patients may instead benefit from active surveillance or upfront RN with dialysis or kidney transplantation listing to avoid renal and extrarenal morbidities following PN [25]. While post-PN progression to ESRD is uncommon in the general population, patients with preexisting low renal function are at risk, with studies estimating an occurrence of over 15%, increasing with tumor size and complexity as nephron preservation becomes limited [25].

Initial univariate analysis revealed several additional comorbidities associated with other perioperative complications which were not different between PN versus RN. Understanding these trends, though not controllable by changing nephrectomy type, is still important, as urologists can anticipate and manage complications. Hypertension was previously demonstrated to influence renal functional outcomes after PN [26]. For example, increased DVT rates with chronic steroid use or PE risk from COPD may suggest need for enhanced VTE prophylaxis in select groups. Similarly, increased MI, bleeds, and UTIs in patients with diabetes may endorse a need for improved cardiac monitoring, antibiotic prophylaxis, and wound closure strategies. Procedure-specific risk calculation indices have been previously proposed, including an algorithm predicting risk of major adverse cardiovascular events following PN based on demographics and baseline factors [27]. Interestingly, this algorithm does not include diabetes, although our study identified diabetes as a significant predictor of MI. Therefore, our analysis indicates the importance of further research to update existing risk calculation algorithms or create new ones where applicable.

Although NSQIP is a high-quality database drawing data from trained institutional reporters, this study has limitations [28,29]. Foremost, its retrospective nature limits interpretation. Moreover, institutional participation in NSQIP is voluntary, and case patterns may not represent all nephrectomies being performed. This study includes data over a one-year period (2019–2020), and findings including surgeon decision-making or patient demographics may have been impacted by the height of the COVID-19 pandemic. Lastly, although 30-day outcomes are a key metric of surgical safety, treatment success also involves long-term outcomes including cancer recurrence and survival, which are not captured by NSQIP. Similarly, imaging results or tumor-specific variables such as nearness to collecting system and overall anatomy, as assessed by RENAL nephrometry scores, are important considerations in surgical assignment, but are not available in NSQIP [7,30,31]. Hence, the findings of this study may only be applied in cases of oncologic equivalence.

## 5. Conclusions

This contemporary analysis demonstrates the value of incorporating case-specific factors within an algorithm to inform T1b-T2 RCC management and patient counseling. Complication rates between PN and RN differed significantly, with higher rates of bleeding, urine leak or ureteric fistulae, renal failure requiring dialysis, and readmissions following PN. However, both techniques remained extremely safe, particularly for patients lacking described risk factors. These findings suggest that increasing PN usage is well-advised, although certain scenarios may warrant risk-based management or RN use.

## Figures and Tables

**Table 1:** Patient characteristics stratified by partial versus radical nephrectomy for renal cell carcinoma.

Baseline Characteristics		All Patients n = 2094	Partial Nephrectomy n = 816 (38.97%)	Radical Nephrectomy n = 1278 (61.03%)	*p*-Value
Age	Mean, SD (years)	61.50, 12.40	60.22, 12.25	62.32, 12.42	**<0.001**
Sex	Female n, %	722	260 (36.01%)	462 (63.99%)	**0.044**
	Male n, %	1372	556 (40.52%)	816 (59.48%)	
Ethnicity	Hispanic	121	44 (36.36%)	77 (63.64%)	0.370
	Not Hispanic	1632	649 (39.77%)	983 (60.23%)	
	Unknown	341	123 (36.07%)	218 (63.93%)	
Race	White	1375	528 (38.4%)	847 (61.6%)	**0.029**
	Asian/ Pacific Islander	64	26 (40.63%)	38 (59.38%)	
	Black	222	105 (47.3%)	117 (52.7%)	
	American Indian/ Alaskan	21	4 (19.05%)	17 (80.95%)	
	Unknown	412	153 (37.14%)	259 (62.86%)	
RCC Stage	T1b	1516	730 (48.15%)	786 (51.85%)	**<0.001**
	T2a	351	56 (15.95%)	295 (84.05%)	
	T2b	178	22 (12.36%)	156 (87.64%)	
	T2, unspecified	49	8 (16.33%)	41 (83.67%)	
Functional Status	Independent	2068	809 (39.12%)	1259 (60.88%)	0.834
	Partially Dependent	23	7 (30.43%)	16 (69.57%)	
	Totally Dependent	2	0 (0.00%)	2 (100.00%)	
	Unknown	1	0 (0.00%)	1 (100.00%)	
Intraabdominal Drain Placement		676	544 (80.47%)	132 (19.53%)	**<0.001**
Peri-operative Antibiotic Use	<24 h	1897	755 (39.8%)	1142 (60.2%)	0.097
	24–72 h	147	47 (31.97%)	100 (68.03%)	
	>72 h	33	10 (30.3%)	23 (69.7%)	
	None	17	4 (23.53%)	13 (76.47%)	
VTE Prophylaxis Method	Mechanical	625	297 (47.52%)	328 (52.48%)	**<0.001**
	Pharmacologic	121	33 (27.27%)	88 (72.73%)	
	Mechanical and Pharmacologic	1293	463 (35.81%)	830 (64.19%)	
	None	55	23 (41.82%)	32 (58.18%)	
Surgical Approach	Open	519	252 (48.55%)	267 (51.45%)	**<0.001**
	Laparoscopic	495	66 (13.33%)	429 (86.67%)	
	Robotic	683	384 (56.22%)	299 (43.78%)	
	Hybrid, Open Assist/ Conversion	397	114 (28.72%)	283 (71.28%)	
Surgical Measures	Mean LOS, SD (days)	3.08, 2.80	3.13, 2.86	3.04, 2.88	0.472
	Mean Operative Time, SD (minutes)	172.08, 75.7	198.45, 72.81	155.24, 72.75	**<0.001**
	Readmissions	117	57 (48.72%)	60 (51.28%)	**0.026**
	Reoperations	37	17 (45.95%)	20 (54.05%)	0.380

Bold text indicates *p* < 0.05.

**Table 2:** Patient comorbidities and peri-operative complications stratified by partial versus radical nephrectomy.

Peri-Operative Characteristics		All Patients n = 2094	Partial Nephrectomy n = 816	Radical Nephrectomy n = 1278	*p*-Value
Medical History	Diabetes	478 (22.83%)	215 (26.35%)	263 (20.58%)	**0.002**
	Smoking	385 (18.39%)	149 (18.26%)	236 (18.47%)	0.905
	Dyspnea	143 (6.83%)	56 (6.86%)	87 (6.81%)	0.961
	Chronic Obstructive Pulmonary Disease	84 (4.01%)	25 (3.06%)	59 (4.62%)	0.077
	Chronic Heart Failure	15 (0.72%)	4 (0.49%)	11 (0.86%)	0.961
	Hypertension	1334 (63.71%)	518 (63.48%)	816 (63.85%)	0.864
	Pre-operative (≤24 h) Acute Renal Failure	4 (0.19%)	2 (0.25%)	2 (0.16%)	0.651
	Dialysis	32 (1.53%)	2 (0.25%)	30 (2.35%)	**<0.001**
	Disseminated Cancer	49 (2.34%)	14 (1.72%)	35 (2.74%)	0.131
	Chronic Steroid Use	67 (3.2%)	25 (3.06%)	42 (3.29%)	0.778
	Bleeding Disorder	60 (2.87%)	20 (2.45%)	40 (3.13%)	0.364
	Mean Pre-operative Creatinine, SD	10.72, 27.70	10.65, 29.63	10.76, 26.41	0.093
Peri-operative Complications	Surgical Site Infections	40 (1.91%)	14 (1.71%)	26 (2.03%)	0.744
	*Superficial Infection*	37 (1.77%)	13 (1.59%)	24 (1.88%)	0.630
	*Deep Wound Infection*	3 (0.14%)	1 (0.12%)	2 (0.16%)	0.841
	Organ/Space Infection	19 (0.91%)	9 (1.1%)	10 (0.78%)	0.451
	Wound Dehiscence	5 (0.24%)	1 (0.12%)	4 (0.31%)	0.383
	Pneumonia	37 (1.77%)	16 (1.96%)	21 (1.64%)	0.591
	Reintubation	13 (0.62%)	7 (0.86%)	6 (0.47%)	0.270
	VTE	26 (1.24%)	13 (1.16%)	13 (1.02%)	0.312
	*Deep Vein Thrombosis*	14 (0.67%)	8 (0.98%)	6 (0.47%)	0.162
	*Pulmonary Embolism*	18 (0.86%)	10 (1.23%)	8 (0.63%)	0.147
	Ventilation > 72 h	9 (0.43%)	6 (0.74%)	3 (0.23%)	0.088
	Acute Kidney Injury	14 (0.67%)	9 (1.1%)	5 (0.39%)	0.051
	Renal Failure Requiring Dialysis	14 (0.67%)	10 (1.23%)	4 (0.31%)	**0.013**
	Urinary Tract Infection	28 (1.34%)	13 (1.59%)	15 (1.17%)	0.415
	Cardiovascular Events	20 (0.96%)	5 (0.61%)	15 (1.17%)	0.252
	*Cerebrovascular Accident*	2 (0.10%)	0 (0.00%)	2 (0.16%)	0.84
	*Cardiac Arrest*	6 (0.29%)	2 (0.25%)	4 (0.31%)	0.777
	*Myocardial Infarction*	12 (0.57%)	3 (0.37%)	9 (0.7%)	0.320
	Sepsis Events	20 (0.96%)	8 (0.98%)	12 (0.94%)	>0.999
	*Sepsis*	16 (0.76%)	6 (0.74%)	10 (0.78%)	0.904
	*Septic Shock*	4 (0.19%)	2 (0.25%)	2 (0.16%)	0.651
	Bleeding Req. Transfusion ≤ 72 h	146 (6.97%)	75 (9.19%)	71 (5.56%)	**0.001**
	Urine Leak or Ureteric Fistula	13 (0.62%)	9 (1.10%)	4 (0.31%)	**0.025**
	Ureteral Obstruction	7 (0.33%)	5 (0.61%)	2 (0.16%)	0.572
	Lymphocele or Lymphatic Leak	36 (1.72%)	17 (2.08%)	19 (1.49%)	0.306

Bold text indicates *p* < 0.05.

**Table 3:** Significant associations between patient co-morbidities and peri-operative complications on univariate analysis.

Complication	Comorbidity (Ref: No Comorbidity)	OR	95% CI	*p*-Value
Wound Dehiscence	Renal failure	175.494	12.591–2446.070	**<0.001**
Pneumonia	COPD	5.789	2.229–15.035	**<0.001**
	Bleeding Disorder	6.596	2.413–18.033	**<0.001**
Reintubation	Bleeding Disorder	5.523	1.089–28.010	**0.039**
VTE	Open (Ref: Minimally Invasive)	2.480	1.121–5.484	**0.025**
	Chronic steroid use	4.245	1.213–14.847	**0.024**
*Deep Vein Thrombosis*	Open (Ref: Minimally Invasive)	3.959	1.325–11.829	**0.014**
	Chronic steroid use	10.048	2.616–38.595	**<0.001**
*Pulmonary Embolism*	COPD	5.212	1.269–21.411	**0.022**
Acute Kidney Injury	Hypertension	7.689	1.008–61.406	**0.049**
Renal Failure Requiring Dialysis	Renal failure	45.734	2.838–736.989	**0.007**
	Open (Ref: Minimally Invasive)	5.859	1.783–19.252	**0.004**
Urinary Tract Infection	Hypertension	3.012	1.013–8.954	**0.047**
Myocardial Infarction	Diabetes	4.988	1.412–17.616	**0.013**
Bleeding Req. Transfusion ≤ 72 h	PN (Ref: RN)	1.524	1.061–2.188	**0.023**
	Open (Ref: Minimally Invasive)	5.070	3.540–7.261	**<0.001**
	Dialysis	3.825	1.547–9.458	**0.004**
	Bleeding disorder	3.105	1.502–6.418	**0.002**
Lymphocele or Lymphatic Leak	Renal failure	12.369	1.117–136.937	**0.040**
	Dialysis	6.680	1.751–25.487	**0.005**
30-day Readmissions	PN (Ref: RN)	1.568	1.062–2.315	**0.024**
	Open (Ref: Minimally Invasive)	1.611	1.080–2.402	**0.019**
	Renal failure	12.734	1.575–102.945	**0.017**
	Chronic steroid use	2.511	1.153–5.471	**0.020**
	COPD	2.646	1.256–5.575	**0.010**
30-day Reoperations	Renal failure	40.017	4.580–349.666	**<0.001**
	Chronic steroid use	4.066	1.364–12.125	**0.012**
	Bleeding disorder	4.256	1.424–12.722	**0.010**

Bold text indicates *p* < 0.05.

**Table 4:** Factors significantly associated with complications upon multivariate adjustment.

Peri-Operative Complications	Associated Comorbidities	Comorbid vs. Non-Comorbid Patients Experiencing Complication	*p*-Value
Wound Dehiscence	Pre-operative renal failure	25.0% vs. 0.19%	**0.010**
Pneumonia	COPD	8.3% vs. 1.5%	**<0.001**
	Bleeding disorder	8.3% vs. 1.6%	**0.004**
Ventilation > 72 h	Bleeding disorder	3.3% vs. 0.3%	**0.026**
Acute Kidney Injury	Hypertension	1.0% vs. 0.1%	**0.024**
Renal Failure Requiring Dialysis	Pre-operative acute renal failure	25.0% vs. 0.6%	**0.027**
Urinary Tract Infection	Diabetes	2.3% vs. 1.1%	**0.043**
	Hypertension	1.8% vs. 0.5%	**0.016**
Bleeding Transfusion	Diabetes	10.5% vs. 5.9%	**0.001**
	Hypertension	8.2% vs. 4.9%	**0.004**
	Dialysis	25.0% vs. 6.7%	**0.001**
	Bleeding disorder	18.3% vs. 6.6%	**0.002**
Urine Leak or Ureteric Fistula	Hypertension	0.9% vs. 0.1%	**0.040**
Lymphocele or Lymphatic Leak	Dialysis	8.3% vs. 1.4%	**0.016**
30-day Readmissions	COPD	11.9% vs. 5.3%	**0.024**
	Pre-operative acute renal failure	50.0% vs. 5.5%	**0.017**
	Dialysis	15.6% vs. 5.4%	**0.030**
	Bleeding disorder	11.7% vs. 5.4%	**0.047**
30-day Reoperations	Pre-operative acute renal failure	50.0% vs. 1.7%	**0.002**
	Dialysis	9.4% vs. 1.7%	**0.018**
	Chronic steroid use	6.0% vs. 1.6%	**0.029**
	Bleeding disorder	6.7% vs. 1.6%	**0.020**

Bold text indicates *p* < 0.05.

## Data Availability

Not applicable.

## Supplementary Materials

The following supporting information can be downloaded at: https://www.mdpi.com/article/10.3390/jcm12010175/s1, Supplementary Table S1. Rates of peri-operative complications stratified by open versus minimally invasive nephrectomy, Supplemental Table S2. Multivariate analysis of associations between patient factors and complications of interest following partial nephrectomy, Supplemental Table S3. Multivariate analysis of associations between patient factors and complications of interest following radical nephrectomy.
