# Supplementary material for: Patient Factors Impacting Perioperative Outcomes for T1b-T2 Localized Renal Cell Carcinoma May Guide Decision for Partial versus Radical Nephrectomy
Source: J Clin Med. 2022 Dec 26;12(1):175. doi: 10.3390/jcm12010175 (PMC9821707; doi:10.3390/jcm12010175)
Supplement: Supplementary file 1 [file jcm-12-00175-s001.zip › jcm-2065736-supplementary.pdf]

Supplementary Table 1. Rates of peri-operative complications stratified by open versus minimally invasive nephrectomy[a].

**Complication**	**Open vs. Minimally Invasive Experiencing Complication**	***p-value***
Surgical Site Infections	2.01% vs. 1.88%	0.856
Superficial Infection	1.82% vs. 1.75%	0.852
Deep Wound Infection	0.18% vs. 0.13%	>0.999
Organ/Space Infection	1.64% vs. 0.65%	0.061
Wound Dehiscence	0.18% vs. 0.26%	>0.999
Pneumonia	2.37% vs. 1.55%	0.256
Reintubation	0.55% vs. 0.65%	>0.999
VTE	2.19% vs. 0.19%	**0.025**
Deep Vein Thrombosis	1.46% vs. 0.39%	**0.014**
Pulmonary Embolism	0.91% vs. 0.84%	0.794
Ventilation > 72 hours	0.36% vs. 0.45%	>0.999
Acute Kidney Injury	0.91% vs. 0.58%	0.378
Renal Failure Requiring Dialysis	1.82% vs. 0.26%	**<0.001**
Urinary Tract Infection	1.82% vs. 1.16%	0.279
Cardiac Events	0.73% vs. 1.03%	0.619
Cerebrovascular Accident	0.18% vs. 0.06%	0.455
Cardiac Arrest	0.00% vs. 0.39%	0.350
Myocardial Infarction	0.55% vs. 0.58%	>0.999
Sepsis Events	1.46% vs. 0.78%	0.198
Sepsis	1.28% vs. 0.58%	0.149
Septic Shock	0.18% vs. 0.19%	>0.999
Bleeding Req. Transfusion ≤72 hours	16.61% vs. 3.56%	**<0.001**
Urine Leak or Ureteric Fistula	1.28% vs. 0.39%	**0.050**
Ureteral Obstruction	0.36% vs. 0.32%	>0.999
Lymphocele or Lymphatic Leak	2.74% vs. 1.36%	0.053
30-day Readmissions	8.03% vs. 4.72%	**0.005**
30-day Reoperations	2.92% vs. 1.36%	**0.023**

Bold text indicates p<0.05.
VTE, venothrombotic embolus
[a]All patients, receiving either partial or radical nephrectomy, were included.

Supplemental Table 2. Multivariate analysis of associations between patient factors and complications of interest following partial nephrectomy.[a]

	Comorbidity (Ref: No Comorbidity)	OR	95% CI	*p-value*
Ventilation	Smoking	N/A	N/A	N/A
	Dyspnea	1.468	0.129-16.729	0.757
	COPD	N/A	N/A	N/A
	Bleeding Disorder	23.962	3.544-162.030	0.001
AKI	Diabetes	0.243	0.030-1.979	0.186
	Smoking	0.540	0.067-4.376	0.564
	CHF	N/A	N/A	N/A
	HTN	5.964	0.734-48.454	0.095
	Pre-operative Renal Failure	N/A	N/A	N/A
	Dialysis	N/A	N/A	N/A
Renal Failure Requiring Dialysis	Diabetes	1.387	0.303-6.340	0.673
	Smoking	0.662	0.081-5.398	0.700
	CHF	N/A	N/A	N/A
	HTN	0.764	0.183-3.192	0.712
	Pre-operative Renal Failure	36.961	1.981-689.607	0.016
	Dialysis	7.323	1.504-35.655	0.014
Bleeds	Diabetes	2.032	1.189-3.470	0.009
	Smoking	0.480	0.210-1.096	0.082
	HTN	1.956	1.036-3.693	0.039
	Pre-operative Renal Failure	5.786	0.338-99.016	0.226
	Dialysis	N/A	N/A	N/A
	Bleeding Disorder	1.715	0.449-6.557	0.430

	Open Approach (Ref: Robotic)	5.237	3.097-8.857	<0.001
Urine Leak or Ureteric Fistula	Diabetes	1.559	0.397-6.116	0.525
	Smoking	1.380	0.281-6.780	0.692
	HTN	3.878	0.457-32.913	0.214
	Pre-operative Renal Failure	N/A	N/A	N/A
	Dialysis	N/A	N/A	N/A
	Open Approach (Ref: Robotic)	2.482	0.654-9.424	0.182
30-day Readmissions	Diabetes	1.137	0.598-2.164	0.695
	Smoking	0.878	0.415-1.857	0.734
	CHF	14.199	1.898-106.213	0.010
	HTN	0.742	0.415-1.325	0.313
	Dyspnea	1.225	0.411-3.657	0.716
	COPD	0.564	0.072-4.416	0.586
	Pre-operative Renal Failure	N/A	N/A	N/A
	Dialysis	N/A	N/A	N/A
	Chronic Steroid Use	0.508	0.066-3.895	0.514
	Bleeding Disorder	N/A	N/A	N/A
	Open Approach (Ref: Robotic)	1.592	0.912-2.779	0.102

[a]N/A denotes variables which were tested but lacked patients fitting the applicable category

Supplemental Table 3. Multivariate analysis of associations between patient factors and complications of interest following radical nephrectomy.[a]

	**Comorbidity (Ref: No Comorbidity)**	**OR**	**95% CI**	***p-value***
Ventilation	Smoking	N/A	N/A	N/A
	Dyspnea	N/A	N/A	N/A
	COPD	N/A	N/A	N/A
	Bleeding Disorder	N/A	N/A	N/A
AKI	Diabetes	3.653	0.605-22.042	0.158
	Smoking	N/A	N/A	N/A
	CHF	N/A	N/A	N/A
	HTN	N/A	N/A	N/A
	Pre-operative Renal Failure	N/A	N/A	N/A
	Dialysis	N/A	N/A	N/A
Renal Failure Requiring Dialysis	Diabetes	8.166	0.807-82.610	0.075
	Smoking	5.202	0.695-38.942	0.108
	CHF	N/A	N/A	N/A
	HTN	0.295	0.031-2.850	0.291
	Pre-operative Renal Failure	N/A	N/A	N/A
	Dialysis	3.598	0.494-26.206	0.206
Bleeds	Diabetes	1.001	0.541-1.854	0.997
	Smoking	0.767	0.387-1.522	0.449
	HTN	1.196	0.685-2.087	0.528
	Pre-operative Renal Failure	N/A	N/A	N/A
	Dialysis	4.709	1.874-11.835	<0.001

	Bleeding Disorder	4.216	1.764-10.078	0.001
	Open Approach (Ref: Robotic)	4.662	2.825-7.691	<0.001
Urine Leak or Ureteric Fistula	Diabetes	N/A	N/A	N/A
	Smoking	N/A	N/A	N/A
	HTN	N/A	N/A	N/A
	Pre-operative Renal Failure	N/A	N/A	N/A
	Dialysis	N/A	N/A	N/A
	Open Approach (Ref: Robotic)	4.067	0.565-29.263	0.164
30-day Readmissions	Diabetes	0.906	0.462-1.778	0.774
	Smoking	0.525	0.226-1.219	0.134
	CHF	N/A	N/A	N/A
	HTN	1.167	0.624-2.182	0.629
	Dyspnea	0.988	1.008	0.378-2.685
	COPD	<0.001	5.403	2.259-12.926
	Pre-operative Renal Failure	N/A	N/A	N/A
	Dialysis	1.863	0.580-5.989	0.296
	Chronic Steroid Use	4.506	1.808-11.229	0.001
	Bleeding Disorder	5.612	2.252-13.983	<0.001
	Open Approach (Ref: Robotic)	1.804	0.998-3.261	0.051

[a]N/A denotes variables which were tested but lacked patients fitting the applicable category
